# Recurrent Disturbances and the Degradation of Hard Coral Communities in Taiwan

**DOI:** 10.1371/journal.pone.0044364

**Published:** 2012-08-30

**Authors:** Chao-Yang Kuo, Yeong Shyan Yuen, Pei-Jie Meng, Ping-Ho Ho, Jih-Terng Wang, Pi-Jen Liu, Yang-Chi Chang, Chang-Feng Dai, Tung-Yung Fan, Hsing-Juh Lin, Andrew Hamilton Baird, Chaolun Allen Chen

**Affiliations:** 1 Biodiversity Research Center/Taiwan International Graduate Program (TIGP), Academia Sinica, Nangang, Taipei, Taiwan; 2 ARC Centre of Excellence for Coral Reef Studies, James Cook University, Townsville, Australia; 3 Institute of Ocean and Earth Sciences, University of Malaya, Kuala Lumpur, Malaysia; 4 National Museum of Marine Biology and Aquarium, Checheng, Pingtung, Taiwan; 5 Graduate Institute of Marine Biodiversity & Evolutionary Biology, National Dong Hwa University, Checheng, Pingtung, Taiwan; 6 Institute of Fishery and Environmental Biology, National Taiwan Ocean University, Keelung, Taiwan; 7 Institute of Biotechnology, Tajen University, Yanpu, Pingtung, Taiwan; 8 Department of Marine Environment and Engineering, National Sun Yat-Sen University, Kaohsiung, Taiwan; 9 Institute of Oceanography, National Taiwan University, Taipei, Taiwan; 10 Institute of Life Science, Chung Hsing University, Taichung, Taiwan; University of Hamburg, Germany

## Abstract

Recurrent disturbances can have a critical effect on the structure and function of coral reef communities. In this study, long-term changes were examined in the hard coral community at Wanlitung, in southern Taiwan, between 1985 and 2010. In this 26 year interval, the reef has experienced repeated disturbances that include six typhoons and two coral-bleaching events. The frequency of disturbance has meant that species susceptible to disturbance, such as those in the genus *Acropora* and *Montipora* have almost disappeared from the reef. Indeed, almost all hard coral species have declined in abundance, with the result that total hard coral cover in 2010 (17.7%) was less than half what it was in 1985 (47.5%). In addition, macro-algal cover has increased from 11.3% in 2003 to 28.5% in 2010. The frequency of disturbance combined with possible chronic influence of a growing human population mean that a diverse reef assemblage is unlikely to persist on this reef into the future.

## Introduction

Coral reefs are declining rapidly on a global scale due to natural and anthropogenic disturbances [1]–[5]. For example, in the Caribbean, coral cover has declined by 80% over the last three decades due to a combination of overfishing, hurricanes, disease and climate induced coral bleaching mortality [6]–[8]. Similarly, at Eilat in the northern Red Sea, the cover of hard and soft corals has decreased from 35% in 1969 to 16.1% in 2001 [9]. Even on the Great Barrier Reef (GBR), one of the better-managed marine protected areas of the world, the mean coral cover among 241 reefs has decreased from 40% in the 1960 s to 20% in the 2000 s [2], [10]. Current estimates suggest that 15% of the world’s coral reefs are severely degraded and projections are that the ecosystem goods and services of 20% of the world’s coral reefs will be lost in 20∼40 years unless more effective management measures are implemented [5].

Coral assemblages respond in a number of ways to disturbance [11]. Coral assemblages can return to the pre-disturbed state, one feature of a resilient ecosystem [12]–[13] For example, coral cover recovered to pre-disturbance levels in less than a year in the Keppel Island, GBR, mainly due to rapid regeneration of remnant coral tissues [14]. In contrast, repeated disturbances to reefs in Tahiti have caused a dramatic change in assemblage structure with many species that dominated the reefs in the 1970 s, such as the *Acropora*, now rare or absent despite coral cover remaining relatively constant [15]–[16]. Finally, multiple disturbances can cause a phase shift [*sensu* 17] from corals towards a degraded state often dominated by macroalgae [6], [11], [18]–[19]. The classical example of a phase shift occurred in Jamaica [19]–[20]. Overharvesting of predatory and herbivorous fishes, led to a reduction in the species richness of grazers and a reduction in predation on *Diadema*, causing a dramatic expansion of the *Diadema* population [19], [21]. Subsequently, a species-specific pathogen caused a significant reduction in the *Diadema* population throughout its geographic range [21]–[22]. Although herbivorous fish populations did respond to the removal of this competitor [22], the grazers could not keep pace with increases in macro-algal abundance. Thick mats of fleshy algae colonized the reef substratum, inhibiting coral recruitment [23].

**Table 1:** The comparison of sampling methods, area, replicate, depth, identification level, and the data used for PCA analysis of each historical data set.

Year	Survey method	Survey Unit	Number ofReplicates	Survey depth	Methods	Identification level	Data set forPCA analysis	Reference
1985	Photo quadrat	4.5 m ^2^	1	10	Colony area was estimate from severalphotographs taken to cover the quadrat.	*Acropora*, *Montipora*, Pocilloporidae,Poritidae, Faviidae, *Heliopora* *coerulea* and other corals. Algaewas not measured	All	[35]
1987 and 1999	Line intercept transect	10 m	25	3 ∼ 23	A transect tape was placed perpendicularto the coast and extended seaward from 3 mdepth to the reef edge at 25 m depth. A 10 mmetal chain was placed parallel to thetransect at 15 m intervals.	Species for Corals Total algae	Only transects between 5 and 10 m depth were used in the PCA	[24], [26]
2003∼2005 2008∼2010	Belt transects	7.5 m^2^	3	5 ∼ 10	Three permanent belt transects wereestablished along depth contours between5 and10 m. Benthic organisms were quantifiedusing 25×25-cm photo-quadrats (120 frames/30 m transect). The percent cover of thebenthic categories was determined usingCoral Count with Excel Extensions vers. 3.6 [42],with 30 random points per quadrat.Surveys were conducted between Marchand May each year.	Species for coralsMacroalgae Turf algae	All	[43]

In this study, changes in hard coral cover and composition on a fringing reef at Wanlitung were analyzed based on surveys conducted from 1985 to 2010. Wanlitung Reef is located on the west coast of Hengchun Peninsula, Kenting National Park (KNP), southern Taiwan, and had a well-developed fringing coral assemblage. However, the reef has suffered from both natural and anthropogenic disturbances, such as typhoons, coral bleaching, overfishing, and sewage discharge, in the last few decades [24]–[32]. In particular, we were interested in whether recurrent disturbance would cause a phase-shift from a coral dominated assemblage to alternative state or whether these reefs might prove resilient.

## Materials and Methods

### Study Site and Historical Disturbances

Wanlitung Reef (21°59.701’N, 120°42.216’E) is part of a fringing reef system along the west coast of the Hengchun Peninsula, southern Taiwan. Facing the junction of the Pacific Ocean (Bashi Strait) and South China Sea, it is influenced by a branch of the Kuroshio Current. Over 280 species of corals have been recorded in the area [33]–[34]. Kenting National Park was established in 1985, the first national park in Taiwan. The aim of KNP was to conserve the terrestrial and fringing reef ecosystems along the Hengchun Peninsula. However, due to a growing population and poor management of human activities the reefs in KNP have been subject to chronic disturbances including overfishing and sewage discharge [30]–[31]. In addition, a nuclear power plant that started to operate in 1985 discharges heated seawater onto the reefs in KNP. In the 26 years of this study, 6 major typhoons have affected Wanlitung Reef: Peggy (category (cat) 5 on the Saffir-Simpson Hurricane Scale [SSHS]) in 1986; Gerald (cat 4) and Lynn (cat 5) in 1987 [21]; Herb (cat 5) in 1996 [26]; Chanchu (cat 4) in 2006; and Morakot (cat 2) in 2009 [32]. In addition, coral bleaching affected Wanlitung Reef in 1998 [26] and 2007 [Kuo and Chen unpubl. Data].

**Figure 1:**
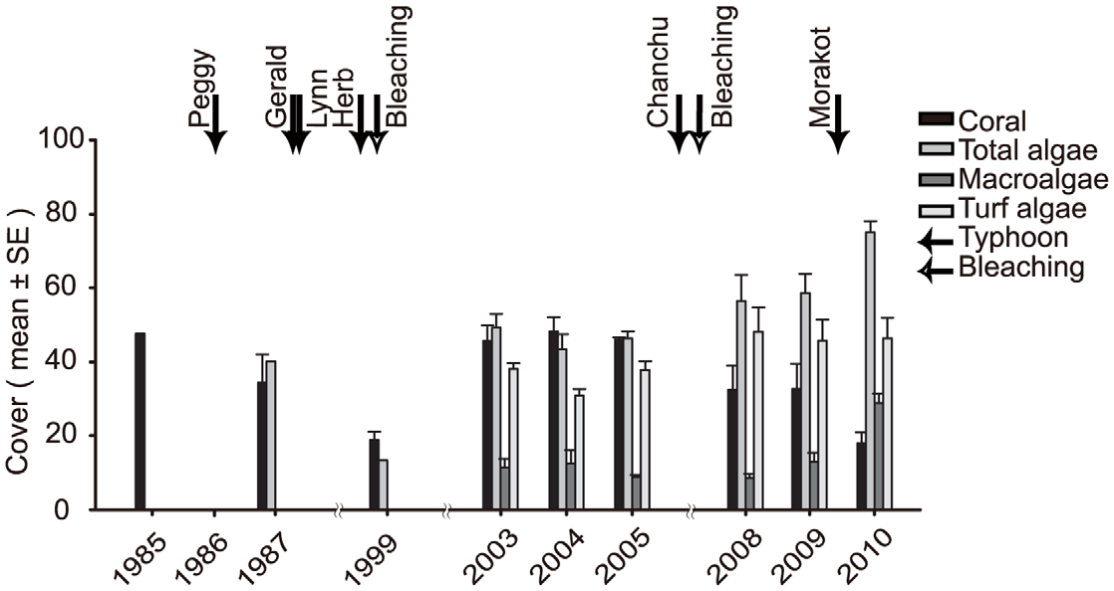
Temporal dynamics of benthic categories at Wanlitung. Temporal dynamics in terms of mean cover (± standard error) of coral, algae (turf and macroalgae) from 1985 to 2010. Typhoons Peggy (1986), Gerald and Lynn (1987), Herb (1996), Chanchu (2006), and Morakot (2009) and two bleaching events (1998 and 2007) are identified.

### Temporal Variation in Benthic Community Structure

Ecological monitoring of the benthic community was conducted at Wanlitung Reef using various techniques to varying degrees of taxonomic resolution in the following years: 1985, 1987, 1999, 2003–2005 and 2008–2010 (see Table 1 for details).

### Statistical Analysis

Variations in the structure of coral assemblages among years were explored using a principal components analysis (PCA). Chang and Jan [35] stated that *Heliopora coerulea* was abundant in Wanlitung in 1985, however, there was no *H. coerulea* in their single quadrat. In order to address this apparent bias in their data, we used the average cover of *H. coerulea* from 1987 to the end of the study to replace the zero value for *H. coerulea* in 1985. For the PCA, data were pooled into seven categories: *Acropora*, *Montipora,* Pocilloporidae, Poritidae, Faviidae, *H. coerulea*, and “other” corals to enable direct comparisons with Chang and Jan [35] (Table 1).

**Table 2:** Mean coverage (%) of all coral species (the top ten species in abundance on each occasion are indicated in bold) at Wanlitung, Taiwan, between 1987 and 2010.

	Mean coverage (%)										
Species name	1985			1987	1999	2003	2004	2005	2008	2009	2010
*Acropora divaricata*				**1.01**	–	–	–	–	–	–	–
*Acropora hyacinthus*				**1.39**	–	–	–	–	–	–	–
*Acropora tenuis*				**0.90**	0.12	–	0.23	–	–		
*Astreopora gracilis*				–	**1.24**	1.19	1.13	0.45	–	0.06	0.01
*Astreopora myriophthalma*				–	0.30	**1.35**	0.83	**1.36**	**1.11**	0.47	**0.79**
*Astreopora randalli*				–	0.02	0.02	–	–	**1.33**	**1.22**	0.16
*Montipora aequituberculata*				0.55	0.25	0.48	**2.73**	**1.92**	0.01	0.17	–
*Montipora informis*				**2.09**	**0.50**	**3.14**	**4.97**	**4.11**	**0.57**	**1.08**	0.14
*Montipora mollis*				–	–	**1.72**	0.72	**1.13**	0.09	**0.84**	0.32
*Montipora monasteriata*				0.55	0.09	**1.71**	**1.31**	**2.11**	0.35	0.41	0.01
*Montipora tuberculosa*				**1.86**	0.03	–	0.01	–	–	–	0.02
*Montipora venosa*				–	–	**3.18**	**4.31**	**4.12**	0.25	0.28	0.07
*Porites annae*				–	**0.68**	0.17	0.36	0.88	0.05	–	–
*Porites lobata*				–	0.36	–	0.08	0.10	0.05	**0.91**	0.25
*Porites lutea*				0.84	0.23	**6.36**	**5.20**	**4.60**	**5.50**	**5.24**	**2.52**
*Porites rus*				0.45	–	0.84	**2.22**	0.77	**0.92**	**1.13**	**0.45**
*Pachyseris speciosa*				0.46	**0.66**	–	–	–	0.05	–	0.03
*Mycedium elephantotus*				**1.04**	0.25	–	–	0.03	0.02	0.16	–
*Echinophyllia aspera*				**1.20**	0.33	0.05	0.01	0.18	0.08	–	–
*Merulina ampliata*				**1.73**	1.25	0.15	0.22	0.11	0.07	0.17	0.36
*Favia speciosa*				0.73	0.22	0.91	**1.42**	0.71	0.47	0.27	0.02
*Favites abdita*				0.73	**0.53**	**1.58**	0.59	0.45	0.01	0.65	**0.78**
*Favites halicora*					0.03	0.18	0.39	0.75	**0.62**	0.30	0.41
*Cyphastrea microphthalma*				0.73	**0.63**	1.21	0.33	0.62	**1.59**	**1.58**	**0.54**
*Platygyra lamellina*				**2.03**	**0.96**	0.50	0.40	0.36	0.05	0.18	**0.52**
*Platygyra pini*				–	**0.79**	0.02	0.01	0.07	0.19	–	0.04
*Turbinaria frondens*				–	0.02	0.92	0.05	**1.69**	0.04	0.16	0.05
*Turbinaria reniformis*				–	–	**1.81**	**2.37**	–	**2.14**	**2.05**	0.44
*Turbinaria stellulata*				–	–	**1.45**	**3.00**	**4.76**	**1.93**	**1.06**	**1.19**
*Heliopora coerulea*				**4.06**	**2.36**	**5.35**	**4.15**	**5.15**	**5.71**	**6.56**	**3.28**
*Millepora exaesa*				–	–	0.43	0.33	0.52	0.37	0.43	**0.48**
*Millepora intricata*				–	–	–	–	–	–	–	**0.51**
Total coral coverage	47.50			35.85	18.50	44.50	46.40	44.48	32.02	31.76	17.69
**Seven major groups used for PCA**											
*Acropora*	23.00			4.98	0.46	0.49	0.46	0.22	0.29	0.17	0.06
*Montipora*	15.00			5.86	2.08	12.67	15.33	13.91	2.42	3.44	1.28
Pocilloporidae	1.50			2.75	0.31	0.35	0.60	0.92	0.42	0.37	0.24
Poritidae	1.00			1.66	1.45	8.35	9.32	7.49	7.56	8.16	3.79
Faviidae	3.50			12.14	7.33	8.25	6.78	5.69	6.36	5.70	4.72
*Heliopora coerulea*	4.85			4.06	2.36	5.35	4.15	5.16	5.71	6.56	3.28
other coral			3.50	4.40	4.51	9.05	9.76	11.11	9.26	7.36	4.32

## Results

### Temporal Variation in Benthic Community Structure

**Figure 2:**
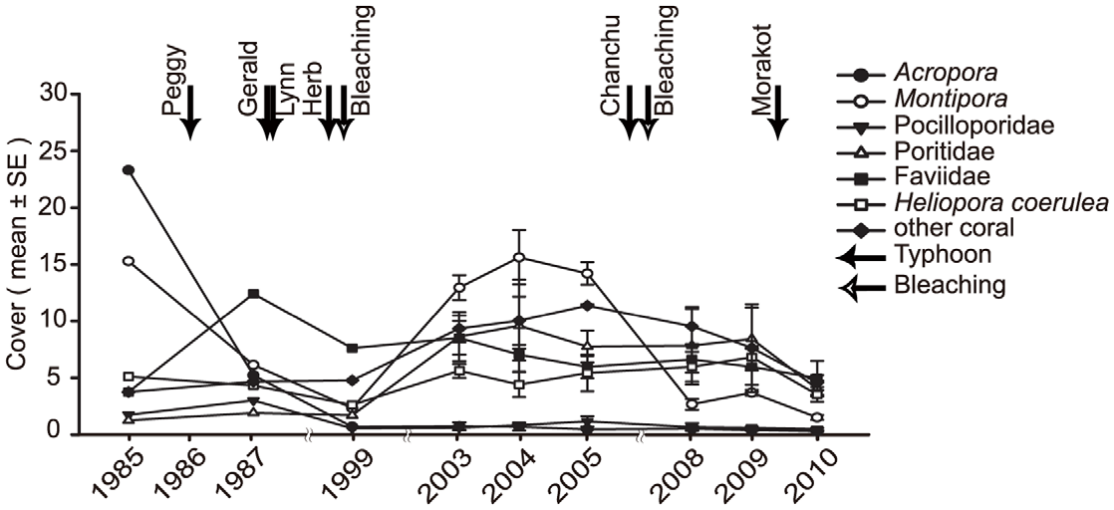
Temporal dynamics of coral community composition at Wanlitung. Temporal dynamics in terms of mean percent cover of eight categories which divided at 1985 from 1985 to 2010. Typhoons Peggy (1986), Gerald and Lynn (1987), Herb (1996), Chanchu (2006), and Morakot (2009) and two bleaching events (1998 and 2007) are identified.

Benthic community structure at Wanlitung has changed considerably over the 26 year period in response to multiple disturbances. Hard coral cover dropped by 63% declining from 47.5% in 1985 to 17.7% in 2010 (Fig. 1). In contrast, total algal cover (macroalgae plus turfs) has doubled between 1987 (the first year it was measured) and 2010 and macroalgal cover increased from 11.3% in 2003 (the first year it was measured independent of turfs) to 28.5% in 2010 (Fig. 1). The decline in hard coral cover was mainly driven by dramatic decreases in the abundance of *Acropora* from 23% cover in 1985 to be almost locally extirpated in 2010 and *Montipora* that declined from 15% to 1.28% (Fig. 2; Table 2). The Faviidae and *H. coerulea* are at similar levels of abundance to 1985, and the Poritidae and other coral taxa have increased in absolute abundance (Fig. 2; Table 2).

Changes in benthic community structure were dynamic with several periods of decline after disturbance and one period of recovery (Fig. 2). Five main periods of change can be identified. Between 1985 and 1987 cyclone Peggy caused a major decline in cover of the *Acropora* and *Montipora* that was partly offset by a 2-fold increase in the cover of Faviidae (Fig. 2). Between 1987 and 1999 multiple disturbances, including three cyclones and a major bleaching event in 1998, led to a highly degraded hard coral assemblage with declines in all taxa except *H. coerulea* and “other” scleractinians (Fig. 2; Table 2). *Acropora* cover declined from 4.9% in 1987 to less than 1% in 1999 and it has not recovered since (Fig. 2; Table 2). 1999–2003 was a period of recovery during which there were no major disturbance, and consequently, most coral taxa, with the notable exception of the *Acropora*, increased in abundance, in particular *Montipora*, *Heliopora* and Poritidae. During this period total coral cover returned to levels first observed in 1985 (Fig. 1; Table 2). Between 2003 and 2005 another period without disturbance led to a period of stasis during which coral cover remained above 40% (Fig. 1, Table 2). Between 2005 and 2010 there has been a steady degradation of the reef in response to bleaching in 2007 and typhoon Morakot in 2009. *Montipora* was particularly affected over this period dropping from 13.9% cover to less than 5% between 2005 and 2008 and cover remained low in 2010 (Fig. 2; Table 2). This gradual degradation in coral cover has also been accompanied by a large increase in the abundance of macroalgae from 8.4% in 2008 to 28.5% in 2010 (Fig. 2).

**Figure 3:**
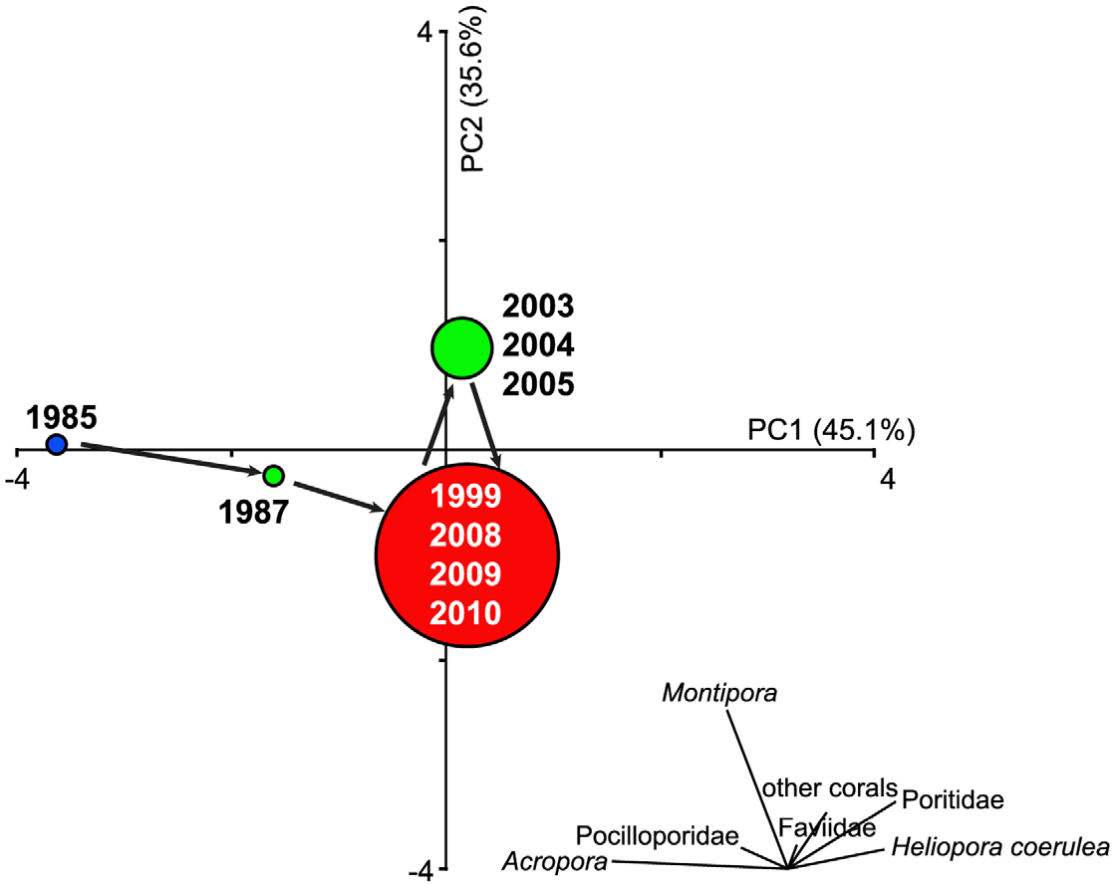
Principal components analysis (PCA) of the coral community structure. With temporal shifts under disturbances of typhoons and bleaching, coral community compositions (with seven major groups) differed in each time period. In addition, community compositions were similar in 1999 and 2008∼2010 after a disaster.

### Temporal Variation in Coral Species Composition

The top 10 coral species in terms of cover on each sampling occasion between 1987 and 2010 are listed in Table 2. *Acropora hyacinthus, A. divaricata,* and *A. tenuis* made up 9.2% of total coral cover 1987, but were reduced to only 1% after bleaching in 1998, and have not recovered since (Table 2). In contrast, *Porites* spp., and *Turbinaria* spp. have increased slightly since 1999 (Table 2). Changes in the abundance of the blue coral, *H. coerulea*, were less dynamic than most other taxa and it was consistently ranked in the top three taxa in terms of abundance (Table 2).

Changes in the relative abundance of coral taxa at Wanlitung from 1985 to 2010 are illustrated in Figure 3. The hard coral assemblages cluster into 4 groups (Fig. 3). In 1985 (blue circle), the original coral community was dominated by *Acropora* and Pocilloporidae. In 1987 (green circle) the community was on the way towards the degraded state (red circle including the assemblages from 1999, 2008, 2009, and 2010) in which all taxa are low in abundance. The assemblages between 2003 and 2005 (green circle) are dominated by *Montipora*, other corals and Faviidae but are distinct from the original 1987 assemblage due to a lack of *Acropora* (Fig. 3).

## Discussion

Recurrent disturbances to the coral reef at Wanlitung have resulted in a degraded coral community in which most taxa, in particular, the structurally important *Acropora*, are less abundant than in 1987. A six year period between 1999 and 2005, during which there were no major disturbances, allowed coral cover to return to 1987 levels. However, the *Acropora* have not been abundant in the assemblage since bleaching in 1998 and the genus is now virtually locally extirpated (Table 2). Furthermore, a 3-fold increase in macro-algal cover since 2008 suggests this trajectory of degradation is unlikely to be reversed. Clearly, the reefs of Wanlitung are not resilient, indeed, the recent trajectory suggests a phase shift towards a depauperate coral assemblage dominated by macroalgae.

Similar declines in coral cover due to natural disturbance have occurred in many other reefs worldwide. For instance, there was a dramatic loss in coral cover as a result of typhoons, from 80% in 1987 to 10% in 1989, on the southern GBR [36]. In the eastern Indian Ocean, coral cover decreased from 48% in 1998 to 11% following bleaching in 1998 [37]. The best understood case of coral degradation is from Jamaica, where coral cover at 7-m depth declined from 75% in 1977∼1979 to 40% in 1980 and then to 5% by 1993 in response to multiple disturbances [19]. The protracted loss of coral cover in Jamaica followed two hurricanes, three bleaching events, a reduction of grazing pressure due to the overfishing and the die-off of *Diadema*, and a potential pulse of nutrients which all contributed to a dramatic increase in algal cover, from 4% in 1977 to 92% in 1993 [19]. However, Dairy Bull, a reef on the north shore near Discovery Bay, Jamaica, is once again dominated by scleractinian corals [38]. Cover at 6∼8 m in 2004 was 54% having doubled since 1995, and the cover of macroalgae had decreased from 45% to 6% possibly as a result of increases in the abundance of *Diadema* [38]. This suggests that even highly degraded reefs can, on occasion, recover.

The recovery of coral cover between 1999 and 2003 was associated with a shift in assemblage structure, similar to Tiahura Reef, Moorea. Recurrent disturbancesat Tiahura, including outbreaks of crown-of-thorns starfish, typhoons, and coral bleaching, have prevented the recovery of many taxa, most notably *Acropora* spp. in Tiahura Reef between 1979 and 2006 [15]–[16]. In Wanlitung these taxa were dramatically reduced firstly by a cyclone in 1986 then bleaching in 1998 and despite 6 years before the next major disturbance neither of these taxa returned and they have not done so since. More recent surveys have found very few *Acropora* recruits on the reefs, suggesting that recruitment failure may also be a factor in the lack of recovery in this taxon although the cause of recruitment failure over such a long period remains unknown.

Turf and macro-algae cover were fairly stable since these taxa were first recorded separately in 2003, however, since 2008 macro-algal cover has increased 3-fold (Fig. 1). In general, herbivores and nutrient enrichment are the two main factors that influence the abundance of macroalgae on reefs [39]. Both may be involved in the recent rise in macroaglae at Wanlitung. Eutrophication is not thought to be a major problem for the reef because nutrients released from human waste in the region are typically trapped by intertidal seagrasses [40] and further diluted by the strong currents at Wanlitung [31]. However, the capacity of the seagrass beds to capture nutrients is limited, and may have recently been exhausted. In addition, the abundance of herbivores is low, because the reefs have been overfished for decades [30]–[31]. The recent high cover of macroalgae may not be having much effect on the corals because its abundance varies seasonally with peaks in the cooler months from November to April followed by a seasonal die off in the warmer months from June to September [41]. Furthermore, macroalgae does not necessarily impede coral recovery. For example, shallow reef communities at Keppel Island, Great Barrier Reef recovered in less a year due to regeneration of remnant coral tissues, very high competitive abilities of the corals allowing them to outcompete seaweed, a natural seasonal decline in a particular species of dominant seaweed, and an effective marine protected area system in the southern Great Barrier Reef [14].

Numerous disturbances over the last 23 years, in particular typhoons and bleaching, have caused long-term changes to coral reef at Wanlitung, however, local stressors such as overfishing, pollution, and coastal development are also likely to have contributed to these changes. Without major changes to current development practices in Kenting, these reefs are unlikely to recover in the near future.
